# Reproductive potential in classical galactosemia: A case series based perspective

**DOI:** 10.1016/j.eurox.2026.100470

**Published:** 2026-06-17

**Authors:** Bianca Panis, Ron JT van Golde, Martijn CGJ Brouwers, Irene MLW Körver-Keularts, Marek J. Noga, M. Estela Rubio-Gozalbo

**Affiliations:** a Department of Pediatrics, Genetic Metabolic Diseases, Mosakids hospital, Maastricht University Medical Centre, Maastricht, the Netherlands; b Department of Obstetrics and Gynaecology, Maastricht University Medical Centre, Maastricht, the Netherlands; c GROW Research Institute for Oncology and Reproduction, Maastricht University, Maastricht, the Netherlands; d Department of Endocrinology and Metabolic Diseases, Maastricht University Medical Centre, Maastricht, the Netherlands; e Cardiovascular Research Institute Maastricht (CARIM), Maastricht University, Maastricht 6200 MD, the Netherlands; f Department of Clinical Genetics, Maastricht University Medical Centre, Maastricht, the Netherlands; g Central Diagnostic Laboratory, Maastricht University Medical Centre, Maastricht, the Netherlands

**Keywords:** Classical galactosemia, Case report, Premature ovarian insufficiency, Spontaneous pregnancy, Fertility counselling

## Abstract

**Introduction:**

Classical galactosemia (CG), caused by galactose-1-phosphate uridylyltransferase (*GALT*) enzyme deficiency, is associated with premature ovarian insufficiency (POI) and subfertility. The last years, a counseling paradigm shift has been advocated with emphasis on subfertility instead of infertility because spontaneous pregnancy can occur.

**Case presentations:**

We describe two women (aged 26 and 30 years) with genetically confirmed CG and POI, who conceived spontaneously. Both adhered to lifelong galactose-restricted diet and had regular endocrine monitoring. Their pregnancies were uncomplicated and each delivered a healthy infant at term.

**Conclusions:**

These cases are in line with the counseling paradigm shift, with natural conception being possible in CG. Reproductive counselling of girls and women with CG should entail fertility preservation and spontaneous pregnancy. In case of desired pregnancy, a period of two years to attempt conceiving should be advised. AMH is not a reliable prognostic parameter for predicting the reproductive potential in CG.

## Introduction

Classical galactosemia (CG, OMIM #230400) is a genetic metabolic disorder caused by deficiency of the enzyme galactose−1-phosphate uridylyltransferase (*GALT*, EC 2.7.7.2012)**.** This enzyme is essential for the metabolism of galactose**,** which together with glucose forms lactose, a disaccharide abundantly present in dairy products.

Infants with CG often show severe symptoms soon after birth when they start consuming milk. Symptoms include feeding problems, jaundice, liver failure, sepsis, hypoglycemia and cataract. After start of strict dietary regime, these symptoms resolve, however long-term complications are common [1].

One of the hallmark long-term complications of CG is premature ovarian insufficiency (POI) in > 90% of women resulting in impaired fertility despite neonatal diagnosis and lifelong dietary galactose restriction. The first paper reporting a 17-year-old female with CG, lacking secondary sexual development and with primary amenorrhea was reported in 1979 [2]. The clinical picture of POI varies from absence of pubertal development to premature menopause [3], [4].

Women with CG often show elevated levels of follicle stimulating hormone (FSH) [5], [6], [7] and decreased levels of anti-Müllerian hormone (AMH) [8] already early in life. AMH is produced by the granulosa cells of early developing follicles but not by primordial or very early primary follicles and has a key function in the regulation of follicular growth and development. AMH levels provide important information about the quantity and quality of the ovarian follicles and reflects ovarian reserve [9]. If primary follicles are activated but undergo atresia before they mature enough to produce AMH, this could explain why AMH levels are extremely low despite some follicle activation [8]. Antral follicle count (AFC) as also ovarian volume [10], [11] are diminished in CG.

Risk factors for the development of POI were initially thought to include homozygosity for the NM_000155.4:c.563 A>G (p.Gln188Arg) variant, elevated levels of galactose−1-phosphate (Gal−1-P), undetectable GALT enzyme activity, elevated levels of FSH, diminished levels of AMH and diminished AFC [11] and severely impaired whole-body galactose oxidation [12] but only spontaneous menarche is currently considered favourable prognostic factor for the likelihood of spontaneous pregnancy in affected individuals [4], [13], [14].

Despite POI, the chance of conceiving spontaneously seems higher in CG than reported in POI due to other cases (5–10%). The reason for this remains unclear [4]. In the literature, about 50 spontaneous pregnancies have been reported since 1971 [14], [15] with success rates ranging from 42.9% to 61% [4] in women trying to conceive in 1–2 years. These insights have led to a shifting in the counseling paradigm for fertility and in case of desirable pregnancy women are advised to attempt to become pregnant naturally for at least 2 years before assisted reproduction is attempted. However, the lack of reliable predictors of fertility, makes it challenging to definitively counsel women about chances of conception.

## Case reports

### Case 1

We report the case of a 26-year-old Dutch woman with CG, diagnosed in the neonatal period and found to be homozygous for the c.563 A>G; p.Gln188Arg mutation in the *GALT* gene. Written informed consent for publication was obtained. She has been on a lifelong galactose-restricted diet since the age of 10 days and has undergone regular endocrine follow-up (Table 1).

**Table 1:** Characteristics of cases.

	Casus 1	Casus 2
Genotype	homozygous for the Q188R mutation in the *GALT* gene	homozygous for the Q188R mutation in the *GALT* gene
Enzyme activity	Not detectable	Not detectable
Neonatal symptoms	Hyperbilirubinemia, two times exchange of blood	Hyperbilirubinemia
Start of galactose restricted diet	Age of 10 days	Age of 6 days
Galactitol and galactose−1-P	At age of 13 years galactitol 90 umol/mmol/creatinine and at age of 17 years 120 umol/mmol/creatinine (normal value <16 umol/mmol creatinine)	At the age of 4 years galactose−1-phosphate 0.3 umol/gHb (normal value <0,20 umol/g Hb) At the age of 17 years galactitol 85 umol/mmol creatinine (normal value <16 umol/mmol creatinine) At the age of 18 years: galactitol 116 umol/mmol creatinine (normal value <13 umol/mmol creatinine)
Pubertal development	Spontaneously, thelarche at age 13 years, menarche at age 14 years	Induced at the age of 12 years
Hormonal evaluation prepubertal		At the age of 1,5 years: FSH 46.8 U/L LH 7 U/L 17-β estradiol < 0.035 nmol/L At the age of 3 years: FSH 50.7 and 54.2 U/L LH 4.2 and 5.5 U/L At the age of 11 years: FSH 45 E/L. LH < 4.5 U/L 17-β estradiol 0.04 nmol/L Start Trisequens
Hormonal evaluation pubertal	Age 13 years: FSH 3.28 E/L LH 3.41 U/L 17-β estradiol 0.27 nmol/L AMH < 0.1 ug/L Age 14 years: FSH 108 U/L LH 60 U/L 17-β estradiol 0.08 nmol/L AMH < 0.1 ug/L: start HRT (ethinylestradiol/levonorgestrel 0.03/0.15 mg) Age 20 years: FSH 46.6 U/L LH 13.3 U/L 17-β estradiol 0.04 nmol/L AMH < 0.1 ug/L Age 24 years after cessation of HRT: FSH 6.9 U/L, 17-β estradiol 0.96 nmol/L Age 25 years: FSH 61.9 U/L, 17-β estradiol 0.4 nmol/L Age 26 years: FSH 5.7 U/L, 17-β estradiol 0.9 nmol/L AMH 0.15 ug/L	Age at 15 years: FSH 40 U/L LH 34 U/L 17-β estradiol 0.33 nmol/L Age at 16 years: FSH 14.5 U/L LH 16.8 U/L 17-β estradiol 0.45 nmol/L At the age of 26 years: FSH 8 U/L 17-β estradiol 0.65 nmol/L AMH < 0.1 ug/L
Imaging	Age 13 years: MRI showed normal uterus, no clear visible ovaries. Age 20 years: MRI showed 1 visible small ovary, diameter 2.5 cm.	At the age of 2 years: ultrasound: normal uturus, no visible ovaries At the age of 15 years: left ovary volume of 2 ml and right ovary of 1 cm, both cystic aspect At the age of 16 years: MRI showed a normal uterus, left ovary: 8*1.4*1.1 cm, right ovary: 0.7*1.3*0.6 cm
Hormonal replacement therapy	Start at age 15 years Stopped at age 23 years	Start at age 12 years Stopped at age 26 years
Pregnancy	At age of 26 years spontaneously	At the age of 31 years spontaneously

AMH: anti Mullerian Hormone: premenopausal 1.0–13.0 ug/L

AMH: anti Mullerian Hormone: postmenopausal < 0.1ug/L

17-beta-Oestradiol:. Follicular phase: 0,11 - 0,37 nmol/L. Luteal phase: 0,26 - 1,10 nmol/L. Post-menopausal: < 0,06 nmol/L

FSH: follicle stimulating hormone

Follicular phase: 2.8–14.4 U/L.

Luteal phase: 1.2 – 9 U/L

Post-menopausal: 21.7–153 Ul/L

LH: Luteinizing hormone

Follicular phase: 1.1–11.6 U/L

Midcyclic phse 17–77 U/L.

Luteal phase: < 0.05 – 14.7 U/L

Post-menopausal: 11.3–39.8 Ul/L

Pubertal development occurred spontaneously, with thelarche at age 13 and menarche at age 14 years. An MRI performed at age 13 demonstrated no visible ovaries. Assessment of antral follicle count (AFC) was not performed. At age 14, hormonal contraception was initiated due to rising FSH levels and low 17-β estradiol levels (Table 1).

At age 24, the patient discontinued hormonal contraception on her own initiative. Surprisingly, she resumed and maintained regular menstrual cycles. Hormonal evaluation at that time revealed FSH 6.9 U/L and 17-β estradiol 0.96 nmol/L indicative of folliculogenesis.

At 26 years of age she conceived spontaneously after 12 months of trying to become pregnant. Her pregnancy was uneventful, and she delivered a healthy male infant at term.

### Case 2

A 31-year-old woman with CG, diagnosed in early infancy and confirmed by genotype as homozygous for the c.563 A>G; p.Gln188Arg mutation in the *GALT* gene. GALT enzyme activity was undetectable. She has been on a galactose-restricted diet since six days of age. Written informed consent for publication was obtained.

At 11 years of age, she was Tanner stage 1 and found to have an elevated follicle-stimulating hormone (FSH) level, indicating POI (Table 1). Puberty was subsequently induced at age 12. At the age of 16 years, MRI abdomen revealed small ovaries. No AFC was assessed.

After temporary stop of hormonal contraception, no menstruation was seen. While on hormonal contraception at age 26, laboratory evaluation further supported a diagnosis of severely diminished ovarian reserve. Fertility counselling was provided, and interfamilial oocyte donation was considered but ultimately not feasible.

At 30 years of age, after 5 years of trying to become pregnant, the patient conceived spontaneously. The pregnancy was uncomplicated. She delivered a healthy female infant at term.

## Discussion

This article reports two women with CG with spontaneous pregnancies despite POI, supporting the paradigm shift of reproductive counseling that encourages women with CG to try to conceive spontaneously for at least two years before assisted reproductive techniques are considered. Like in these two women, POI is characterized by a heterogeneous and unpredictable course. Despite the overall decline in ovarian reserve, intermittent follicular activity may still be observed, and residual follicles can occasionally produce viable oocytes This suggests that ovarian dysfunction in CG does not invariably equate to absolute follicular depletion [3].

The cause of POI in CG is unknown, but studies suggest that folliculogenesis is impaired already at very young age, leading to decreased ovarian function and POI [16], [17], [18]. Limited data show that the number of primordial follicles rapidly decline in early years [19], [20], [21]. A case report described normal ovaries at the age of 7 years but hypoplastic ovaries in the same girl at the age of 17 years [22]. Normal histology was observed in two neonates, whereas reports of females at age ranging from 16 to 26 years, describe that there was either none or only a few primordial follicles with absence of mature follicles, suggesting a maturation arrest [19], [20], [23], [24], [25], [26], [27], [28], [29].

Disruption of critical signalling pathways involved in folliculogenesis—particularly PI3K/AKT, mitogen-activated protein kinase (MAPK), Insulin-like Growth Factor 1 (IGF−1) pathways—has been observed in both CG patients and animal models [30], [31]. These pathways are essential for follicle growth, survival, and activation, and their dysregulation in the galactosemic state contribute to follicular arrest or loss. Downstream factors such as TGF-beta and GDF−9 are also implicated in impaired follicle development. In *GalT* gene-trapped (*GalT*KO) mouse models, early activation and subsequent depletion ("burnout") of primordial follicles mimic the progressive ovarian failure seen in CG patients [32]. In recent transcriptomic studies, ovarian tissue from girls with CG displayed extensive alterations in gene expression related to endoplasmic reticulum stress, oxidative stress, and DNA damage responses [31]. Single-nucleus RNA sequencing localized these changes to granulosa cells and oocytes, revealing suppression of PI3K/AKT signaling and upregulation of pro-apoptotic transcripts. Consistent with these human data, GALT-deficient mice exhibited reduced phosphorylated EIF2α levels, premature follicle activation, and follicular loss that could be partially rescued by pharmacologic ISR modulation [5]. Together, these transcriptomic findings emphasize dysregulated cellular stress responses as key drivers of ovarian insufficiency in CG.

Abnormal glycosylation, another feature of CG, may interfere with hormone (FSH)-receptor interactions, although studies are inconclusive about its direct role in POI [10], [33].

Despite low or undetectable AMH levels—typically indicative of diminished ovarian reserve, but not in CG—some of these patients experienced spontaneous puberty, aligning with prior findings that spontaneous pubertal development occurs in CG. Although anti-Müllerian hormone (AMH) is commonly used as a marker of ovarian reserve in clinical practice, its reliability in the context of CG is limited. AMH is secreted by granulosa cells of growing pre-antral and small antral follicles, but not by primordial or very early primary follicles. In CG, ovarian dysfunction is believed to interfere with early folliculogenesis, leading to primary follicles that do not transition adequately and undergo cell death. Consequently, AMH levels are often undetectable or markedly low, even in the presence of a preserved pool of primordial follicles. This disconnect means that low AMH values in CG patients may reflect disrupted follicle development rather than true follicular depletion. Accordingly, spontaneous puberty and even spontaneous pregnancy have been reported in individuals with undetectable AMH, indicating that AMH alone is not a reliable predictor of residual ovarian function or reproductive potential in this population [34].

The phenotypic variability observed among individuals with CG, particularly in relation to ovarian function and reproductive outcomes, represents the influence of other genetic and environmental modifier effects. Despite sharing the same *GALT* variants, patients often differ significantly for example in the number of primordial follicles they are born with that comprises a wide range, in their pubertal development, AMH levels, and in their capacity for spontaneous pregnancy. This variability implies that factors beyond the primary *GALT* genotype contribute to disease expression. Potential modifiers may include polymorphisms in genes involved in folliculogenesis, hormonal signaling, or cellular stress responses. Environmental influences, such as early-life nutrition or metabolic control, may also play a role. Further research is needed to elucidate these modifiers, which could ultimately refine prognostic assessments and allow for more personalized approaches to managing fertility in individuals with CG.

## Conclusion

These cases highlight the importance of realistic and sensitive fertility counselling, tailored endocrine follow-up, and individualized patient care. It is important that physicians emphasize the occurrence of spontaneous pregnancies in women with CG and therefore a time-window of at least 2 years for attempting to conceive naturally should be advised before fertility preservation or oocyte donation will be started. The POI in CG is likely a process involving a cascade of events triggered by cellular distress (ER and oxidative damage) and impaired signalling pathways, leading to early follicle activation and death (apoptosis/autophagy/other) with rapid follicle depletion, and subsequent early ovarian dysfunction and subfertility. AMH alone is not a reliable predictor of residual ovarian function or reproductive potential in this population. Potential modifiers and environmental influences are likely to play a role. Further research is needed to elucidate these modifiers, which could ultimately refine prognostic assessments and allow for more personalized approaches to managing fertility in individuals with CG.

## Key takeaways

• Spontaneous pubertal development in CG has emerged as a favourable prognostic factor for future fertility. This highlights that ovarian insufficiency is not an absolute state and that some degree of residual function may persist into adolescence and early adulthood.
• Approximately 50 spontaneous pregnancies have been reported in the literature. Success rates among those actively trying to conceive range from 42.9% to 61% within 1–2 years, emphasizing that spontaneous ovulation and conception are achievable, albeit unpredictable.
• Ovarian reserve markers often underestimate fertility potential. Although AMH is widely used to estimate ovarian reserve, its utility in CG is limited. AMH does not reflect the presence of primordial follicles, which may still exist in CG despite undetectable hormone levels. Thus, AMH should not be used as a sole predictor of reproductive potential in this population.
• In women with CG desiring pregnancy, temporary withdrawal of HRT can be considered to assess for endogenous ovarian activity. A conception attempt period of up to two years is supported by literature, during which ovulatory activity should be monitored.

## Trial registration

Not applicable.

## Attestation statements

• The subjects in this trial have not concomitantly been involved in other randomized trials (not applicable).
• Data regarding any of the subjects in the study has not been previously published unless specified.
• Data will be made available to the editors of the journal for review or query upon request.
• CARE checklist was followed.

## CRediT authorship contribution statement

**van Golde Ron:** Writing – review & editing, Visualization, Conceptualization. **Rubio-Gozalbo M.:** Writing – review & editing, Supervision, Conceptualization. **Noga Marek:** Writing – review & editing, Conceptualization. **Brouwers Martijn:** Writing – review & editing, Conceptualization. **Körver-Keularts Irene:** Writing – review & editing, Conceptualization. **Bianca Panis:** Writing – original draft, Data curation, Conceptualization.

## Funding

Not applicable.

## Conflict of interest statement for all authors

Authors have no conflict of interest.

## Declaration of Competing Interest

The authors declare that they have no known competing financial interests or personal relationships that could have appeared to influence the work reported in this paper.

## Data Availability

Not applicable.
